# Dried Blood Spots and Plasma Separation Cards can Broaden Access to Molecular Testing for HBV, HCV and HIV

**DOI:** 10.1002/rmv.70059

**Published:** 2025-08-04

**Authors:** Jeffrey V. Lazarus, Neil Parkin, Huma Qureshi, Benjamin LaBrot, John W. Ward

**Affiliations:** ^1^ CUNY Graduate School of Public Health and Health Policy New York New York USA; ^2^ Barcelona Institute for Global Health (ISGlobal) Barcelona Spain; ^3^ Data First Consulting Sebastopol California USA; ^4^ Doctor's Plaza Clifton Karachi Pakistan; ^5^ Roche Molecular Systems Pleasanton California USA; ^6^ Coalition for Global Hepatitis Elimination Task Force for Global Health Decatur Georgia USA

## Abstract

Approximately 3.5 million people acquired HBV, HCV, or HIV‐1 in 2022, and 340 million persons are infected with at least one of these three viruses, mostly in low‐ and middle‐income countries (LMICs). Nucleic acid amplification tests (NAAT) are precise and sensitive tools for viral infection diagnosis, monitoring and sequencing. NAATs typically rely on plasma specimens, which can be challenging to collect, store and transport in LMICs, limiting the reach of NAAT testing in rural and other resource constrained settings where trained phlebotomists and/or a cold chain for preservation and shipping are not available. Alternative specimen types that can overcome this limitation include dried blood spots (DBS) and dried plasma prepared using the Cobas Plasma Separation Card (PSC). We performed a literature review of DBS and PSC use for NAAT of HBV, HCV, or HIV‐1 to summarise their performance characteristics, relative advantages and disadvantages and potential to support expanded access to NAAT. DBS have been used extensively for HIV‐1 diagnosis and viral load monitoring, as well as for HBV and HCV, albeit to a much lesser degree. Compared to plasma, DBS perform well in terms of accuracy but have lower sensitivity. There is a risk of low specificity due to the presence of cellular nucleic acids in DBS and a resulting over‐estimation of viral load. The PSC has similar accuracy and sensitivity compared to DBS, but improved specificity due to the removal of cellular components. Both DBS and PSC have the potential to enhance access in populations where the use of plasma is challenging.

**Abbreviations**
AASLD: American Association for the Study of Liver Diseases
CROI: Conference on Retroviruses and Opportunistic Infections
DBS: dried blood spots
EASL: European Association for the Study of the Liver
EID: early infant diagnosis
HBsAg: HBV surface antigen
IAS: International AIDS Society
LMICs: low‐ and middle‐income countries
NAAT: nucleic acid amplification tests
PoC: point‐of‐care
PSC: plasma separation card
PWID: people who inject drugs
VL: viral load
WHO: World Health Organisation

## 1 Introduction

The World Health Organisation (WHO) estimates that a combined 3.5 million people were newly infected with HBV (1.2 million), HCV (1.0 million) or HIV‐1 (1.3 million) in 2022, and that over 340 million people worldwide are infected with at least one of these three viruses, most of whom live in low‐ and middle‐income countries (LMICs) [1, 2]. Infection with HBV, HCV or HIV has serious individual and public health consequences [3, 4] and WHO and other international and national public health institutions have established ambitious goals for elimination of these viruses as global health threats [1, 5, 6]. In 2022, the percentage of the population living with these viruses who were diagnosed was estimated to be 13% for HBV, 36% for HCV, and 86% for HIV‐1 [1, 7, 8, 9]. Therefore, identification of those who are living with the virus but not yet diagnosed is an international health priority.

The last few decades have seen advances in technology that support detection and quantification of viral nucleic acids in blood specimens. Nucleic acid amplification tests (NAAT) that mostly rely on polymerase chain reaction and real‐time detection and quantitation have become powerful and sensitive tools for three broad purposes: (1) diagnosis of active or persistent infection, for example in prevalence surveys; (2) viral load (VL) measurement to monitor treatment effectiveness, disease staging/progression, or therapeutic decision‐making; and (3) sequencing of viral genomes for subtyping, drug resistance assessment or molecular epidemiological studies. All three of these applications have been brought to bear for HBV, HCV and HIV‐1, albeit to different extents. Clinical practice guidelines include use of NAAT as part of diagnostic testing algorithms for all three viruses [7, 8, 9].

**FIGURE 1:**
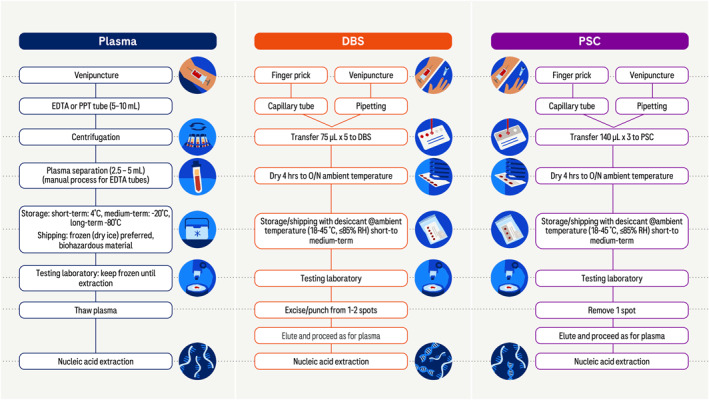
Comparison of steps for preparation of plasma, DBS and PSC. The steps required for specimen preparation when NAAT is indicated are shown. Both DBS and PSC can be prepared using capillary blood (e.g., form a finger prick) or venous blood following venipuncture. Nucleic acids extracted from DBS will contain relatively more DNA (illustrated as a double helix) versus RNA (single stranded). EDTA, ethylenediaminetetraacetic acid; PPT, plasma preparation tube; DBS, dried blood spot; O/N, overnight; PSC, plasma separation card.

The preferred biological specimen type for NAAT testing is blood plasma or serum, from which viral nucleic acids are extracted. Proper collection, handling, storage and shipment of plasma involves several steps (Figure 1). Each of these steps has environmental condition requirements (e.g., limits on temperature, time, and humidity) to preserve the integrity of nucleic acid(s). For example, plasma must be frozen for long‐term storage to prevent genomic nucleic acid degradation during storage and shipping. Furthermore, phlebotomy should only be performed by a trained technician using the necessary equipment in an appropriate setting, and transfer of potentially infectious materials from one tube to another should take place under strict biosafety procedures. These requirements cannot be met in many locations, due to paucity of trained personnel, lack of reliable electricity supply for biosafety cabinets, refrigerators and freezers, and limited availability of dry ice for shipping frozen specimens. This restricts the reach of diagnostic testing to settings that can support the required steps and excludes many rural settings in LMIC where phlebotomists and/or a cold chain for preservation and shipping are not available. Thus, reliance on plasma specimens for NAAT represents an important barrier to reaching the epidemic control goals set forth by the WHO and other agencies.

In addition to resource constraints in LMIC, the requirement to attend a phlebotomy facility can be an obstacle in certain populations in resource‐rich countries, such as the rurally located, unhoused, and people who inject drugs (PWID). For such populations, there can be significant losses in follow‐up when multiple clinic visits are needed. For this reason, WHO and others have recommended decentralised reflex testing for HBV and HCV, a two‐step diagnostic algorithm whereby a positive rapid antibody test is followed by NAAT using the same specimen or a second one collected at the same visit [8, 9].

There are several ways to overcome the problems associated with use of plasma in LMIC and other populations. One is to move away from the central laboratory model through the use of point‐of‐care (PoC) or near‐PoC platforms for NAAT. These have been reviewed elsewhere [10, 11, 12].

A second approach is to use alternative specimen types that obviate the need for specialised equipment and cold storage. Many such solutions have been developed, to varying extents, but the most widely used and well‐characterised is dried blood spots (DBS). Recently, the Cobas Plasma Separation Card (PSC; Roche Molecular Systems, Pleasanton, CA, USA) has become available for collection of dried plasma from blood specimens for detecting and quantifying HIV‐1 and HCV RNA. While other options exist, none are currently being manufactured and commercialised, although some may be useful in research settings (see Table 1).

**TABLE 1:** Alternative specimen types in addition to DBS.

Product	Manufacturer	Publication(s)^a^	Website
Cobas plasma separation card	Roche	Carmona [13]; Vubil [14]	https://diagnostics.roche.com/global/en/products/params/cobas‐plasma‐separation‐card.html
FTA filter paper	Many	Yacouba [15]; Li [16]	Na
Hemaspot	Spot On sciences	Brooks [17]; Manne [18]; Bunthen [19]	https://www.spotonsciences.com/products/
Mitra (hemaPEN)	Neoteryx	—	https://www.neoteryx.com/collection‐kits
Plasma separation card	Telimmune	—	https://www.telimmune.com/plasma‐separation‐cards
RNASound RNA sampling card	FortiusBio	Lau [20]; Levine [21]	https://www.fortiusbio.com/products/perforated‐rna‐sampling‐cards
Tasso + kit	Tassoic	Rosenblum [22]	https://www.tassoinc.com/tasso‐plus‐kit
ViveST	ViveBIO	Levine [21]; Diallo [23]; Zanoni [24]	Na^b^
VLPlasma	Burnet Institute/Nanjing BioPoint diagnostics	Pham [25]	https://www.burnet.edu.au/research/projects/plasma‐separator‐device‐for‐hiv‐viral‐load‐testing‐vlplasma/

^a^ Example publications or conference abstracts reporting use of the method/device for NAAT of HBV, HCV, HIV‐1, or other RNA virus.

^b^ No longer in operation, product not available.

This narrative review will summarise the performance characteristics, relative advantages and disadvantages of DBS and PSC for NAAT of HBV, HCV and HIV‐1, and their potential to support expanded access to NAAT.

## 2 Methods

We searched PubMed (from inception to January 21, 2025) for journal articles using the following search strings:

- DBS: ((dried blood spots [Title/Abstract] OR dried blood spot [Title/Abstract] OR DBS [Title/Abstract] OR dried blood [Title/Abstract] OR dry blood [Title/Abstract]) AND (hepatitis [Title/Abstract] OR HBV [Title/Abstract] OR HCV [Title/Abstract] OR HIV [Title/Abstract] OR human immunodeficiency virus [Title/Abstract]))
- PSC: ((plasma) AND (“separation”)) AND (card)

We also searched for conference abstracts from 2019 to 2024, to the extent possible given barriers to free access. Conferences searched included the Conference on Retroviruses and Opportunistic Infections (CROI), International AIDS Society (IAS), European Association for the Study of the Liver (EASL), and the American Association for the Study of Liver Diseases (AASLD). Conference archive websites listed below were searched for abstracts with ‘dried blood’ or ‘DBS’ or ‘plasma separation’ in the title. Abstracts referencing NAAT for HBV, HCV or HIV‐1 in the title were examined further.

- CROI (2019–2024): https://www.croiconference.org/search‐abstracts/
- IAS (2019–2024): https://www.abstract‐archive.org
- EASL: (2019–2024): Supplements in *Journal of Hepatology* https://www.journal‐of‐hepatology.eu
- AASLD: Supplements in *Hepatology* https://journals.lww.com/hep/pages/issuelist.aspx

Literature searches were performed by NP. Exclusion criteria were agreed upon by all authors.

### 2.1 Exclusion Criteria

Publications and abstracts were excluded from consideration if they met one or more of the following criteria:

- Protocol description, case report (single subject), editorial or review article including meta‐analyses
- No relevant data included, or methods unclear
- Non‐standard DBS (i.e., not Whatman 903 or Munktell paper) or Cobas PSC
- Not used for HBV, HCV or HIV‐1 NAAT; for example, DBS used for drug level measurement (typically of tenofovir in the context of HIV‐1 pre‐exposure prophylaxis), antibody or antigen testing
- Conference abstract or pre‐print that was subsequently published
- Not relevant for other reason(s)

## 3 Results

**FIGURE 2:**
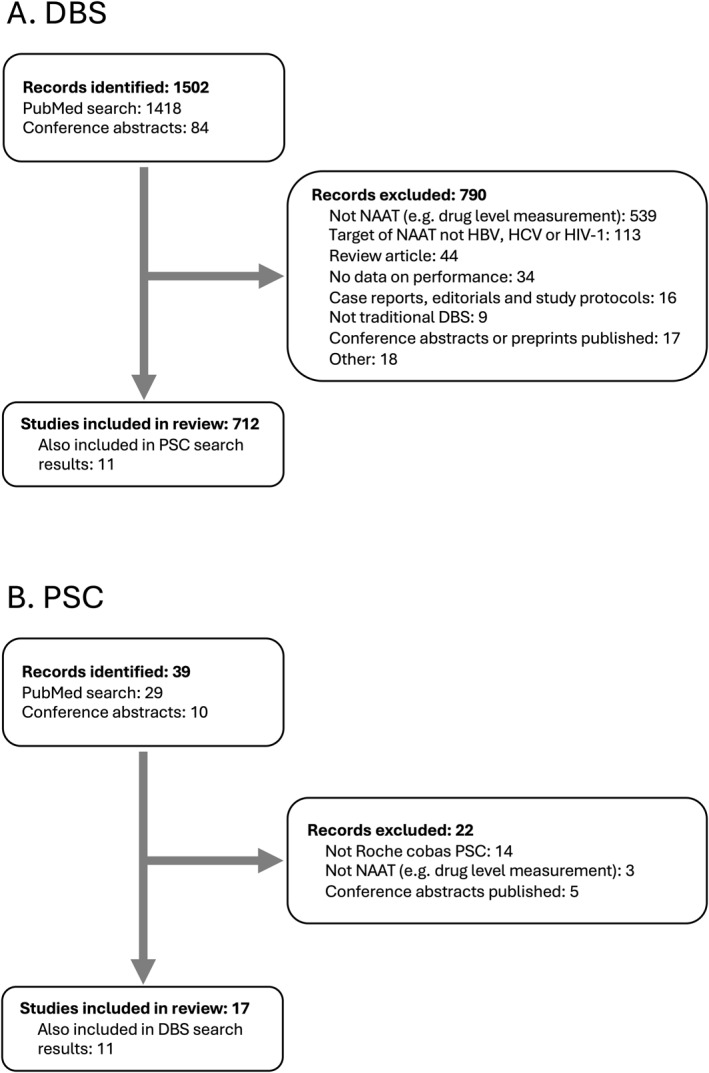
Literature search results for DBS (A) and PSC (B). Additional details related to the reasons for exclusion can be found in the Supporting Information S1. DBS, dried blood spot; NAAT, nucleic acid amplification test; PSC, plasma separation card.

The DBS search resulted in 1395 published articles and 84 conference abstracts (see Figure 2). Of these, 774 were excluded. The majority (561 of 705, 79.6%) of the remaining publications included HIV‐1, 127 included HCV, and 52 included HBV (several articles included more than one virus). The purpose of testing was diagnosis in 352 publications (e.g., early infant diagnosis [EID] for HIV‐1), VL measurement in 225 publications, and sequencing for drug resistance or phylogenetic studies in 202 publications (several articles included more than one purpose; see Table 2).

**TABLE 2:** Publication counts.

Virus	Purpose	DBS	PSC
HIV	Diagnosis	261	1
	Viral load	174	8
	Sequencing	167	1
HBV	Diagnosis	27	1
	Viral load	21	1
	Sequencing	18	0
HCV	Diagnosis	94	2
	Viral load	40	5
	Sequencing	22	0

*Note:* (numbers do not sum to number of articles due to overlap i.e., multiple specimen types, viruses or purposes in one article).

The PSC search resulted in 28 published articles and 10 conference abstracts; 22 publications were excluded (Figure 2). The majority (10 of 16, 62.5%) of the remaining articles included HIV‐1, six included HCV, and two included HBV (two articles included HBV and HCV). The main purpose of testing in 13 publications was VL measurement, diagnosis in three, and sequencing for drug resistance in one (one article included more than one purpose; see Table 2).

A complete list of literature search results is available as online Supporting Information S1.

### 3.1 Dried Blood Spots

**FIGURE 3:**
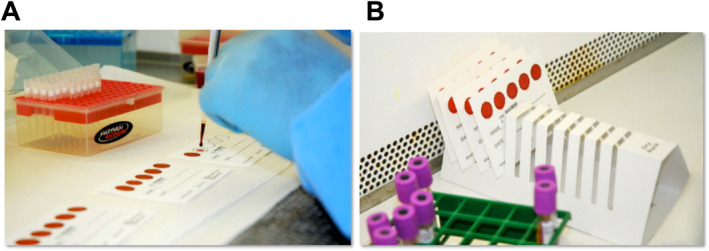
Examples of DBS preparation by pipetting (A) and drying in a rack (B). Photographs taken by NP in June, 2011 at the UVRI in Entebbe, Uganda [26].

DBS are made using blood from a finger or heel prick that is spotted directly or transferred with a capillary pipette on filter paper (usually Whatman 903 or equivalent), then dried by exposure to the air. They can also be made using anticoagulated venous blood and pipetting (Figure 3), if venipuncture is an option. Once dry, viral nucleic acids are temporarily stabilised and can be shipped to the testing site at ambient temperature. For optimal stability, especially of viral RNA, packaging in air‐tight bags with desiccant is recommended. The DBS (or a portion of it) are then excised or punched out and nucleic acids extracted in the laboratory. For long‐term storage, DBS need to be frozen at −20°C or (preferably) −80°C in plastic bags with desiccant.

#### 3.1.1 Advantages of DBS Versus Plasma

The main advantages provided through use of DBS, when prepared from a finger or heel prick, are that a trained phlebotomist and a centrifuge are not required, the specimen can be collected at the point of care or even in the patient's home, and short‐term storage and shipping at ambient temperature is possible, so that refrigeration or freezing is not needed. This can greatly enhance the feasibility of specimen collection in certain populations, and may also reduce costs associated with specimen collection, storage and shipping.

#### 3.1.2 Disadvantages of DBS Versus Plasma

Although more stable than in liquid plasma, viral RNA in DBS is subject to degradation if the filter paper is exposed to high temperature or humidity for extended periods of time. The precise limits of time, temperature, and humidity are dependent on each downstream application and need to be determined empirically.

Importantly, it is almost certain that tolerated conditions are less stringent when DBS will be used for protein‐ (e.g., HBV sAg or antibody) or DNA‐ (e.g. HIV‐1 EID) based assays than when they will be used for RNA detection or quantification. This has important procedural implications in settings where reflex testing is being considered (discussed below).

The volume of blood per spot is limited to approximately 75 μL, roughly corresponding to a plasma volume of 40 μL. This represents a significant reduction in the quantity of plasma used for nucleic acid extraction, with a resulting increase in the lower limit of detection. The magnitude of the increase depends on the number of DBS spots (or portions thereof, e.g. when small punches are excised) used and the volume of plasma used for comparison ‐ typically between 0.1 and 1.0 mL.

Since the DBS contains white blood cells in addition to plasma, intracellular viral nucleic acids may be present in the extracted material. This has the potential to result in quantitative over‐estimation, or false positive qualitative results (if the plasma VL is just under the detection threshold), compared to RNA extracted from plasma; this phenomenon had been well described for HIV‐1 [27]. The degree to which DNA contributes to the test result is dependent on the relative quantity of RNA and DNA in the specimen, and on selectivity (DNA vs. RNA) of the extraction and detection methods. For HIV‐1 VL, these considerations have been described previously [28]. The problem can be mostly mitigated through use of methods that remove the cellular components, or that selectively extract or amplify and detect the viral RNA versus DNA from DBS.

#### 3.1.3 Evidence on Effectiveness in Closing the Diagnostic Gap

Studies that demonstrate the utility of DBS in expanding the reach of NAAT fall into two categories:

1. Technical evaluation or validation of the performance of DBS for NAAT, usually compared to plasma. These studies serve to help establish optimal laboratory procedures, demonstrate proof‐of‐concept, and characterise sensitivity and specificity.
2. Implementation studies performed in LMICs, remote settings, and special populations that illustrate how use of DBS supports improved access and/or impact on healthcare equity.

In general, methods for nucleic acid extraction, detection, quantitation and sequencing are well‐described. Technical performance studies often show a reduction in sensitivity (higher limits of detection or higher assay failure rate at low VL ranges) compared to plasma, as expected based on the lower volumes of plasma in DBS than are usually processed when using separated plasma. However, reduced sensitivity may be offset by the increased access made possible by reduced reliance on a cold chain as described above.

Below are some examples of publications, grouped by infecting virus, that highlight how DBS enable access to NAAT in areas where reliance on plasma is challenging.

##### 3.1.3.1 HBV

Stene‐Johansen et al. [29] performed a cohort study in which DBS and plasma were collected from 26 HBV‐positive participants in a hospital setting in Addis Ababa, Ethiopia. DBS were dried then stored in plastic bags with desiccant at room temperature for between four and 39 days before shipment to Oslo, Norway for testing. HBV DNA levels were quantified by real‐time RT‐PCR (Abbott). HBV DNA was detectable in all 23 DBS with plasma VL over 500 IU/mL, and two of five with lower VL. HBV VL measurements from DBS and plasma were highly correlated with each other, although VL from DBS was nearly 4‐fold lower than from plasma. In a controlled stability sub‐study using DBS from three participants, there was no significant decrease in VL for up to 12 weeks of storage at ambient temperature.

Toyé et al. [30] collected DBS from 613 HIV‐1 infected children in 72 health centres and regional hospitals in Senegal. DBS were transported to laboratories in Senegal where HBV VL and sequencing for drug resistance analysis was performed if HBsAg was detected (*n* = 25). HBV VL was detectable in 18 specimens, and sequencing was completed in 15 of these. Comparison with plasma was not performed.

Vinikoor et al. [31] compared HBV VL measured using DBS or plasma, collected from 68 HIV‐1 infected, HBsAg‐positive adults attending two public sector clinics in Lusaka, Zambia. DBS prepared from capillary blood (finger prick) were dried at ambient temperature for one to three days, then transported to a central laboratory where they were frozen at −80°C for one to six months. HBV VL measurements (Roche) from DBS and plasma were highly correlated with each other, although VL from DBS was approximately 40‐fold lower than from plasma.

##### 3.1.3.2 HCV

Aponte‐Melendez et al. [32] determined HCV prevalence in 150 PWID in Puerto Rico including people living in rural areas and unhoused persons, using street‐based outreach and respondent‐driven sampling. DBS were prepared from capillary blood (finger prick) and shipped to New York for testing. HCV RNA was detected in 79 of 109 anti‐HCV antibody positive DBS specimens using an assay with a detection limit of 250 IU/mL (Hologic).

Pedro et al. [33] surveyed 443 young (18–29 years old) PWID in New York City using venue‐based street outreach and chain‐referral (peer‐to‐peer) sampling methods. Eligible participants visited an urban study site where DBS were prepared and rapid HCV tests performed. HCV RNA was detected in 64 of 110 anti‐HCV antibody positive DBS specimens, 57 of which were sequenced for genotyping and phylogenetic analysis.

##### 3.1.3.3 HIV‐1

EID for HIV‐1 using DBS, based on detection of HIV‐1 DNA by PCR, has been part of WHO guidelines since 2016 [34]. The use of NAAT and DBS in LMIC for EID is necessary because of the potential for maternal anti‐HIV‐1 antibodies to interfere with traditional serologic testing of infants less than 18 months old. The performance of DBS for EID is robust, and has been reviewed extensively elsewhere [35, 36]. The qualitative nature of this testing, and the fact that DNA rather than the more fragile RNA is targeted, help to make this application relatively straightforward.

More recently, DBS have been adopted as a specimen type for HIV‐1 VL measurement, with the main purpose of monitoring the success of anti‐retroviral treatment [10, 27, 35]. For this application, care must be taken to avoid over‐quantification of HIV‐1 RNA in DBS because of the presence of cellular nucleic acids that can also contribute to the VL result, as described above. DBS are used for routine HIV‐1 VL monitoring in Vietnam [37] and several countries in Africa [38, 39, 40].

The use of DBS as a specimen type for sequencing HIV‐1, mostly in order to assess anti‐retroviral drug resistance, is commonplace in public health surveillance settings. Detailed methods and recommended collection, storage and shipping conditions have been summarised by the WHO [41]. Several surveys of pre‐treatment or acquired HIV‐1 drug resistance in LMIC using DBS as the specimen type have been implemented [42]. As for VL measurement, prolonged exposure to high heat or humidity leads to degradation of viral RNA and increased sequencing assay failure [26].

### 3.2 Plasma Separation Card

**FIGURE 4:**
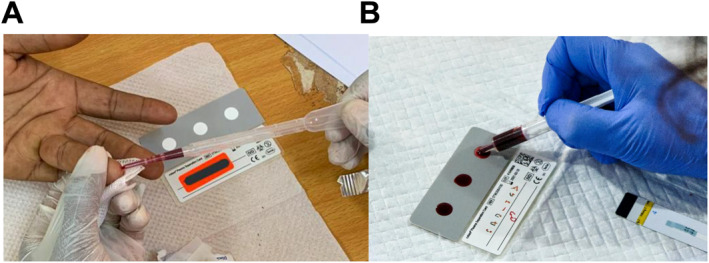
Examples of PSC preparation by transfer pipette (A) and spotting with a syringe (B). Photographs taken by Dr. Camila Picchio, Barcelona Institute for Global Health (ISGlobal), Hospital Clinic, University of Barcelona, Barcelona, Spain and used with permission.

The Cobas PSC was designed to support specimen collection for HIV‐1 RNA testing in contexts where use of plasma is not possible, and to overcome some of the drawbacks of using DBS for HIV‐1 VL measurement, including reduced specificity. Blood is applied to the PSC (Figure 4) and is filtered in situ by capillary action, transferring the liquid phase (plasma) to a second sheet of membrane that is then dried. A stabiliser in the second membrane preserves the integrity of viral nucleic acids to support ambient temperature storage and shipping; minimal effects on HIV‐1 VL were observed for up to 21 days at 18°C to 45°C and up to 85% humidity [13, 14].

#### 3.2.1 Advantages of PSC Versus Plasma

All of the advantages listed above for DBS compared to plasma also apply to PSC.

Because of the physical filtration step, blood cells are removed, and the resulting material more closely mimics liquid plasma compared to DBS. Therefore, the dangers of over‐quantification and false positive results related to the presence of cellular nucleic acids in DBS are not of concern with the PSC.

#### 3.2.2 Disadvantages of PSC Versus Plasma

The recommended volume of blood applied to the PSC is 140 μL per spot, resulting in a limitation related to sensitivity compared to established procedures for plasma that use more than about 75 μL of plasma.

#### 3.2.3 Evidence on Effectiveness in Closing the Diagnostic Gap

The publications summarised below highlight the technical performance of PSC compared to plasma or demonstrate field performance and potential to expand access to NAAT.

##### 3.2.3.1 HBV

Picchio et al. evaluated the acceptability and feasibility of PSC for HBV diagnosis in Cameroon and Uganda [43]. Feasibility (as judged by the percentage of PSC with at least one spot properly filled) was very high (99%) in Uganda, but only moderate (53%) in Cameroon. A similar trend was observed with respect to patient acceptability and ease of sample collection, with much more favourable reporting observed in Uganda compared to Cameroon. Possible explanations for the difference between countries proposed by the authors included differences in health care worker experience or training.

Martínez‐Camprecios et al. [44] prepared PSC from capillary blood from anti‐HBV or ‐HCV antibody positive patients attending outpatient hospital clinics in Spain and Angola. PSC were shipped in regular mail from Angola to Spain where they were processed for analysis. HBV DNA and HCV RNA were assayed. Amongst HBsAg‐positive samples from Spain, 62% had detectable HBV DNA in plasma compared to 33% in PSC. Most of the negative PSC samples were from patients with low levels of HBV DNA (below 2000 IU/mL). HBV and HCV viral loads from PSC and plasma were tightly correlated with each other, but average PSC VL was 12‐ to 40‐fold lower.

##### 3.2.3.2 HCV

Qureshi et al. collected PSC and paired plasma specimens for HCV VL testing at two sites in Pakistan [45]. The sites where participants were sampled and PSC prepared differed in terms of the level of supporting infrastructure. At the first site, comprising community health centre outpatient clinics, VL results were well‐correlated with each other. However, at the second site, in an underserved, resource‐limited, semi–urban area, VL from PSC were poorly correlated with the matched plasma sample. Possible reasons for the difference between sites proposed by the authors included differences in training or performance of health care workers, or storage and/or transport conditions.

##### 3.2.3.3 HIV‐1

The PSC was originally developed for the specific application of HIV‐1 RNA VL measurement, given an issue with the use of DBS for this purpose, especially in LMIC (see above) [13, 46]. PSC have been evaluated for HIV‐1 VL monitoring in Mozambique [14], South Africa [47] and Uganda [48]. Compared to plasma, performance of HIV‐1 VL assessment using a 1000 copies/mL threshold (the WHO‐recommended level used to define treatment failure) was high, with sensitivity ranging from 87.5% to 100% and specificity from 87.9% to 99.3% (Table 3). Carmona et al. [13] and Chimoyi et al. [47] also assessed user acceptability and found that the vast majority of users reported a high usability score.

**TABLE 3:** Performance of PSC for HIV‐1 viral load.

First author, year	Country	Specimen types evaluated	VL assay	PSC preparation	N patient samples	Sensitivity, 1000 copies/mL	Specificity, 1000 copies/mL	bias (PSC‐plasma), log copies/mL
Carmona 2019 [13]	South Africa	PSC, plasma	Cobas HIV	Capillary blood	485	97.0%	97.2%	0.05
Vubil 2020 [14]	Mozambique	PSC, DBS, plasma	CAP/CTM	Capillary blood	609	99.8%	97.3%	0.065
Vubil 2020 [14]	Mozambique	PSC, DBS, plasma	CAP/CTM	Venous blood	609	100.0%	98.2%	0.067
Kiyaga 2023 [48]	Uganda	PSC, DBS, plasma	Cobas HIV	Capillary blood	185	92.9%	87.9%	0.12
Kiyaga 2023 [48]	Uganda	PSC, DBS, plasma	Cobas HIV	Venous blood	185	89.0%	96.6%	−0.14
Chimoyi 2024 [47]	South Africa	PSC, plasma	CAP/CTM	Capillary blood	446	87.5%	99.3%	nd

The feasibility of PSC for HIV drug resistance (sequencing) was assessed in a small‐scale comparison of plasma, DBS and PSC [49]. This evaluation is relevant in laboratories that might perform drug resistance sequencing as a reflex test following determination of VL over 1000 copies/mL, an indicator of treatment failure, in settings where PSC are used as the specimen type for VL. Nearly all (97%) of the samples tested supported resistance test completion.

## 4 Discussion

DBS and PSC are similar in many ways, but they also have several distinguishing characteristics. Similarities include:

- Both can overcome the obstacles associated with the collection, storage and shipment of plasma specimens through reduced reliance on phlebotomy and refrigeration. This has the potential to improve access to molecular diagnostic testing in areas of the world where these obstacles are prevalent. These areas also often have high infection incidence and prevalence. Therefore, the use of DBS or PSC to collect specimens for NAAT have high potential for impact on healthcare equity.
- Both can be prepared from finger or heel prick blood, or by pipetting anticoagulated, venous blood collected by phlebotomy. The choice between these options will likely be driven by the availability of trained phlebotomists and equipment, and whether venous blood is needed for other purposes.
- Optimal results require operator training to ensure recommended procedures are followed. As described above, good performance is usually achieved in controlled laboratory settings, but in real‐world situations it can be more variable and likely dependent on operator training and experience.
- Preservation of nucleic acids requires use of desiccant to minimise exposure of the dried material to humidity in the air. This requirement is likely to be more important for HCV and HIV‐1, whose detection and measurement in plasma is primarily based on RNA rather than DNA.
- Detection thresholds are higher than for plasma because of the reduced volume of plasma in the dried spot that is used in the extraction step, compared to already‐separated plasma. However, lower analytical sensitivity may be considered an acceptable trade‐off in exchange for increased access to diagnostic testing, because use of DBS or PSC can enable identification of infected people that would otherwise not be found.

Key differences between DBS and PSC are highlighted in Table 4. The most significant differences are that the material used for nucleic acid extraction from DBS includes cellular components, while for PSC it more closely resembles plasma. This feature eliminates the potential for cellular DNA or RNA to contribute to the NAAT result. Also, the PSC contains stabilisers that maintain the integrity of RNA, making VL under‐estimation or false negative results less likely compared to DBS. Therefore, PSC may be expected to have higher tolerance to mishandling that may occur as a result of delays in shipping or operator error.

**TABLE 4:** Distinguishing features of DBS versus PSC for NAAT.

Attribute	DBS	PSC	Notes
Blood volume per spot	50–75 µL	140 µL	For DBS, two spots can be processed per extraction to increase sensitivity
Number of spots per card	5	3	Maximum number per card
Cellular component removed	No	Yes	
RNA stabiliser	No	Yes	
Cost (card only)	∼$1	∼$5	Total estimated cost per HIV‐1 VL test $19 (DBS) to $23 (PSC) [50].
Shipping conditions	Ambient < 14 days^a^	≤ 21 days at 18°C–45°C	PSC can tolerate up to 85% humidity
Storage conditions	Ambient < 14 days^a^	Ambient < 56 days	
Utility demonstrated for:			
Diagnosis	HBV, HCV, HIV‐1	No	
Viral load	HBV, HCV, HIV‐1	HBV, HCV, HIV‐1	PSC validated for HCV and HIV‐1
Sequencing	HBV, HCV, HIV‐1	HIV‐1	DBS validated for HIV‐1

^a^ Ambient = 18°C–30°C. Combined storage and shipping time must be less than 14 days for HIV‐1 drug resistance testing (sequencing) [41].

Another important consideration and likely difference between DBS and PSC is cost. As a product developed for a specific purpose, with associated manufacturing quality control, the PSC itself is more costly compared to Whatman 903 filter paper used to make DBS. However the use of PSC leads to more accurate results, and when analysed as the cost of a VL test per correct result, the total estimated cost differences were minimal [50].

Achieving the ambitious goals for hepatitis elimination set out by WHO will require overcoming several obstacles, including technical ones related to increasing access to testing for persons in hard‐to‐reach areas of the world. Improving access to diagnostic tests to identify infected individuals and monitor their response to treatment must involve decreased reliance on collection, storage and shipping of plasma. While DBS have been used for longer and methods are well‐established, its imperfections leave room for improvement. Some of these drawbacks have been addressed with the PSC.

As with any new technology or method, there are challenges associated with implementation of DBS or PSC in the context of existing healthcare delivery systems. Health care workers (especially those in remote field settings) need to be trained on all technical steps of new procedures while adapting existing infrastructure and logistics to support optimal specimen preparation and handling, which can be very context‐dependent. It is also important to involve clinicians, non‐governmental organisations, government and public health agencies, and policymakers in adopting these strategies to support innovative models of healthcare.

While DBS have been used for NAAT for many years, especially for HIV‐1, the PSC is a more recently developed product, and several questions have not yet been addressed thoroughly. Therefore, future research should focus on the following areas:

- Use of PSC for sequencing, such as for drug resistance testing, which is often performed using remnant specimens collected for VL measurement. The optimal storage conditions and limitations in time and temperature may be less forgiving than for VL, since the amplicons used for drug resistance sequencing are usually longer than for VL, thus making template fragmentation more detrimental. While an early conference report is encouraging [49], larger studies that incorporate a wider range in VL and extraction methods are warranted.
- Use of PSC for antigen (e.g., HBV S, core or E antigen) or antibody testing, which may be more accessible or less costly than NAAT.
- Self‐collection that is patients preparing DBS or PSC in their own home using a kit [51, 52, 53, 54, 55, 56].
- Operational research to guide implementation of PSC in effective models of care that expand access to hepatitis diagnosis and treatment.

This review has some limitations. Studies demonstrating poor performance of DBS or PSC for NAAT might be less likely to be published in peer‐reviewed journals, possibly resulting in biased assessments of performance. Since the PSC is a commercial product that has been available for a much shorter period of time compared to DBS, the number of studies that evaluated PSC is much lower; this could reduce the opportunity for publication of studies evaluating performance. This is not a systematic review, and the publications highlighted as examples may not be representative of all published data; given the heterogeneity in laboratory methods used with DBS, as an uncontrolled product and procedure, a systematic review would be a significant challenge.

Our literature review provides a window into relatively under‐studied aspects of the use of DBS or PSC for NAAT in the context of managing care for HBV, HCV, and HIV‐1 infected persons. These include cost effectiveness compared to use of plasma, especially in LMIC and other difficult to reach populations, larger‐scale comparisons between PSC, DBS and plasma for HBV and HCV, the use of PSC for EID of HIV‐1, and use of PSC for reflex testing, that is PSC as a specimen type for antibody testing followed by retrieval of remnant spots from the same card for NAAT. For reflex testing, it is crucial that differences in requirements for specimen preservation and contamination are considered, since the first test (usually an antibody test) uses a more stable analyte. Since the storage requirements for antibody testing are less stringent than those for RNA‐based NAAT, the specimen must always be treated as if it will be used for NAAT until it has been determined whether the RNA NAAT test will be needed. This applies to both temperature and humidity control as well as the potential for inter‐specimen molecular contamination.

In conclusion, DBS and PSC are feasible alternatives for specimen collection that overcome constraints related to reliance on plasma as the specimen type for NAAT to diagnose and monitor HBV, HCV, and HIV‐1 infections. Each alternative has advantages as well as technical limitations. Additional operational research and sharing of lessons learnt will guide the choice of DBS and PSC for diverse settings and target populations. With this growing body of experience, DBS and PSC based NAAT testing will simplify viral testing and care helping countries reach goals for HBV, HCV and HIV elimination.

## Author Contributions

**Jeffrey V. Lazarus:** conceptualisation, methodology, writing – review and editing. **Neil Parkin:** data curation, methodology, visualisation, writing – original draft preparation, writing – review and editing. **Huma Qureshi:** conceptualisation, writing – review and editing. **Benjamin LaBrot:** conceptualisation (lead), data curation, funding acquisition, methodology, visualisation, writing – review and editing. **John W. Ward:** conceptualisation, writing – review and editing.

## Supporting information

Supporting Information S1
